# Laryngeal tuberculosis mimicking a vallecular cyst: a diagnostic pitfall

**DOI:** 10.1590/0037-8682-0241-2026

**Published:** 2026-10-16

**Authors:** Elif Gozgec, Emre Ozil

**Affiliations:** 1 Ataturk University School of Medicine, Department of Radiology, Erzurum, Turkey.

A 59-year-old male patient presented to the otorhinolaryngology clinic with hoarseness and difficulty swallowing that had persisted for approximately seven months. Laryngoscopic examination revealed a mass in the vallecula region at the oropharyngeal level. Pathologic examination of a 1-cc biopsy sample taken from this mass revealed findings consistent with granulomatous diseases, including tuberculosis. Magnetic resonance imaging demonstrated an 18 × 11 × 20 mm cystic lesion in the left vallecula, T2-hyperintense, T1-hypointense, with peripheral enhancement and increased diffusion (Figure 1). Multiple cervical lymph nodes with cortical thickening and diffusion restriction were also noted. Ultrasound revealed punctate calcifications in the left level III lymph nodes (Figure 2A). Interferon-gamma release assay and QuantiFERON-TB test results were positive. A subsequent chest computed tomography (CT) scan performed for screening purposes revealed changes in sequelae suggesting a history of tuberculosis (Figure 2B). Anti-tuberculous therapy was initiated.

**FIGURE 1:**
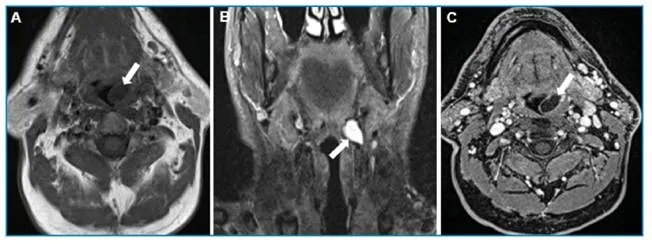
On magnetic resonance imaging, a cystic lesion (arrows) is observed in the left vallecular region: it appears hypointense in the T1-weighted axial image **(A)**, hyperintense in the T2-weighted coronal image **(B)**, and shows peripheral enhancement in the post-contrast axial series **(C)**.

**FIGURE 2:**
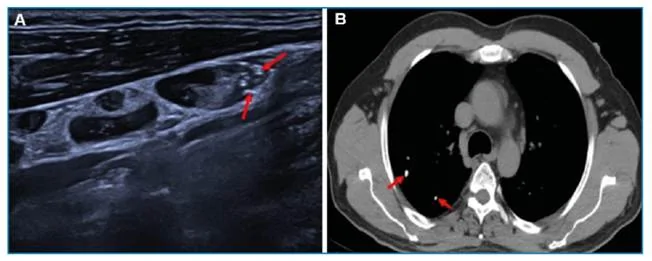
In the neck ultrasound image **(A)**, a lymph node with calcifications (arrows) is visible. In the non-contrast axial soft-tissue window chest CT image **(B)**, punctate calcifications (arrows) are visible in the right lung.

Laryngeal TB may present as an isolated, mass-like lesion with minimal or absent pulmonary findings. It is frequently confused with laryngeal carcinoma (cancer) due to shared symptoms including hoarseness, dysphagia, and mass-like lesions detected via laryngoscopy^1^ ^,^ ^2^. These shared symptoms create a diagnostic dilemma that may delay establishment of the correct diagnosis, even in regions where tuberculosis remains endemic^1^. Such atypical presentations should be kept in mind, particularly in endemic regions, and imaging should play a supportive role in diagnosis.

## References

[1] Pandiyan H, Sivanand N, Sathish Kumar S (2022). Isolated Laryngeal Tuberculosis: a Diagnostic Dilemma. Indian J Otolaryngol Head Neck Surg.

[2] Yammouri Z, Chaouche S, Alami G, Harmouch F, Alami B, Lamrani Y (2025). Laryngeal Tuberculosis Mimicking Laryngeal Carcinoma: A Case Report. Cureus.

